# Injection 3D Printing of Doubly Curved Ceramic Shells in Non-Synthetic Particle Suspensions

**DOI:** 10.3390/ma17163955

**Published:** 2024-08-09

**Authors:** Vesela Tabakova, Christina Klug, Thomas H. Schmitz

**Affiliations:** Department of Visual Arts, Faculty of Architecture, RWTH Aachen University, 52062 Aachen, Germany; tabakova@kg.rwth-aachen.de (V.T.); klug@kg.rwth-aachen.de (C.K.)

**Keywords:** LDM, 3D clay printing, injection 3D printing, suspension

## Abstract

This paper examines the application of non-synthetic particle suspensions as a support medium for the additive manufacturing of complex doubly curved ceramic shells with overhangs between 0° and 90° using clay paste. In this method, the build-up material is injected within a constant volume of air-permeable particle suspension. As the used clay paste does not solidify right after injection, the suspension operates like a support medium and enables various print path strategies. Different non-synthetic suspension mixtures, including solid and flexible components such as quartz sand, refractory clay, various types of wood shavings, and cotton flocks, were evaluated for their ability to securely hold the injected material while allowing drying of the water-based clay body and its shrinkage. The balance between grain composition, added water, and the compressibility of the mixture during printing and drying played a pivotal role in the particle suspension design and assessment. Furthermore, the moisture absorption of the particle suspension and the structural integrity of the layer bond of the fired ceramics were also assessed. The examined additive manufacturing process not only enables the production of meso-scale doubly curved ceramic shells with average overhang of 56° but also introduces a new practice for designing specialized surfaces and constructions.

## 1. Introduction

Ceramics are being rediscovered in the construction industry as a particularly durable and sustainable alternative to concrete materials. Doubly curved ceramic shells could allow for the incorporation of bespoke elements for structural applications, e.g., in the field of façade cladding. Such geometries can incorporate aesthetic, performance, and installation features that are produced in a single operation. Although the Additive Manufacturing (AM) process enables precise modeling of large-format components, the Liquid Deposition Modeling (LDM) [1] that is used for the production of soft clay bodies is not fully developed yet. LDM utilizes viscous kaolinite clay paste that solidifies several hours after the printing process. After drying, the printed green bodies have to be sintered to reach their optimal structural strength. Although the programmed data contains precise instructions for the movements of the actuator, there is no possibility in the process to influence the viscous 3D-printed clay body and its complex material behavior after deposition [2]. As a result, deviations between the planned model and the realized 3D print are to be expected, particularly evident in the case of material overhangs and perforations. Based on the type of clay and viscosity of the paste, the overhang of a geometry is limited to around ≥30° from the vertical plane, otherwise a deviation from the digital model is to be expected [3]. The limitation of geometrical freedom for potential designs due to material properties is an obstacle to the true incorporation of LDM as a production method for structural parts, such as those in the construction sector [4].

If one investigates the LDM process in more detail, it can be seen that, due to the comparatively fast and inexpensive process and the vast deposits of clay worldwide, further approaches have already been explored to remedy the challenge with deformation of 3D-printed clay bodies, which are not only concerned with different recipes of ceramic pastes, as is frequently the case [5,6]. Starting with considerations on the material application method and extrusion path modeling for ceramic pastes [7], up to approaches for the utilization of qualitative characteristics of pastes [8,9]. A technological development for the extrusion method has already been achieved in which the clay body is specifically stabilized or can be transformed with the help of thread insertions within the extrusion body [10]. As LDM in 3D printing systems with fixed actuators also entails technical limitations for the production of doubly curved components, the screw-based extrusion method is supplemented through robotic systems. These robotic fabrications have the advantage of being able to angle the actuator with 6 degrees of freedom (DOF) and, furthermore, can be used in combination with different types of custom fixed or adjustable molds [11,12]. Such approaches are mainly used in the area of flat curved components [13]. Different bulk materials have been hitherto used as substrate 3D printing molds, especially for the production of concrete lattice shells [14,15]. However, even when using 6 DOF robotic arms, it does not prevent the limitations in shape freedom caused by the employed substrate beds as molds. Due to the shrinkage behavior of the used pastes and the substrate hold angle, this process can be only applied to produce shallow doubly curved panel-like elements. Although clay material is also suitable as a support material for complex component geometries, this must be removed after production, at great expense, and this has a very different effect on the surface structure of such components.

There are technologies available on the market that allow the production of ceramic components with steep overhangs trough the supporting effect of surrounding particles. However, it will be seen that these methods are not particularly suitable for the production of ceramic parts in context of the building industry. Direct Ink Writing (DIW) is a process similar to LDM; however, the ceramics pastes in DIW are mostly used with chemical binders for high technical applications, while the LDM pastes are water-based and are widely used in construction industry. The scale and speed of LDM allows the production of components on a meso-scale, while DIW can be used for producing smaller elements [16]. It will become apparent that the rheological properties [17], interparticle interaction [18], and the influence of scale will play a significant role in the production of LDM components [19,20]. Binder Jetting (BJ) is widely used to produce porous, doubly curved, or high-resolution ceramic parts by building the 3D model in a dry powder substrate that can simultaneously act as a support and construction material for the 3D-printed object [21]. In this process, a binder is applied onto a ceramic powder bed and a new powder layer is distributed on top of the previous one by a roller. The binder is used to hold the geometry until the sintering process, during which the binder is burned and the ceramic object reaches its final structural strength. However, this approach is limited and allows for the production of parts with size ranges from few millimeters up to a maximum of 800–1800 mm [22]. Another process that uses layer-by-layer binding of an aggregate is Selective Paste Intrusion (SPI), where aggregate particles are bonded by a cement paste [23]. In this process, the binder provides the structural strength of the object and the object reaches its maximum structural strength after the curing of the cement. The second technology mentioned here could potentially be applicable to meso-scale components in clay pastes in the future but would require further consideration in terms of material and machines. The kaolinite clay paste used in this examination does not harden with the aid of additional chemical binders, as would be necessary for the SPI.

The issue of the fabrication of overhangs in AM can be dealt with in another technical system, named injection 3D printing. This process utilizes a supporting medium in the form of a suspension gel, which allows production of components with steep overhangs [24,25]. This process is not only investigated on a micro-scale but is also used in structural elements in the building industry. However, and this is important, the use of liquid building materials in connection with injecting in a gel suspension can only be used in combination with materials using chemical binders. For this reason, only chemically hardening pastes, like concrete, could be used for this method. Due to the material properties, water-based clay pastes cannot dry through evaporation inside non-air-permeable gels. Injection 3D printing in quartz sand has already been investigated. In this process, a fast-curing concrete mixture was also use [26]. The investigation showed that a fine dry aggregate could also be a suitable material for injection 3D printing. Only two of the projects mentioned above focused on producing complex geometries that consist of continuous doubly curved surfaces. This is likely attributable to the inherent challenges in achieving structural integration while accounting for material parameters, rheological behavior, and the interlayer binding, which complicate the production process of complex-shaped components. But in addition to design elements with openings, primarily closed surfaces are needed as cladding elements in an architectural context.

In the context of the existing LDM technology and by advancing the capabilities in the area of injection 3D printing within a constant volume of non-synthetic particle substrate, this study aims to open new possibilities to produce complex, customized, doubly curved ceramic shell components targeting the whole range of overhang angles, without considering the modeling of support structures, which would have to be removed later at great expense. This innovative approach aligns with the growing demand for personalized and environmentally friendly building solutions.

## 2. Materials and Methods

### 2.1. Experimental Set-Up with Liquid Deposition Modeling System

**Figure 1:**
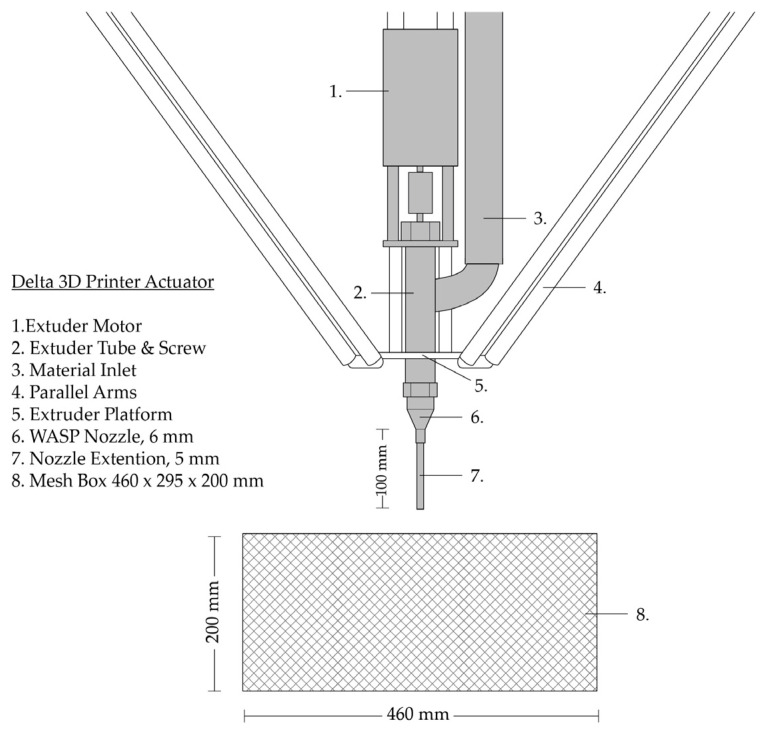
Delta 3D Printer Actuator.

The experimental objects were produced using a Delta WASP 40100 clay 3D printer (Massa Lombarda, Italy) equipped with an LDM extruder. This delta-style printer operates with 3 DOF, enabled by a parallel kinematic mechanism comprising three arms connected to vertical linear carriages. To change the printer’s position within the Cartesian coordinate system, all three arms must be adjusted simultaneously. The printer has a cylindrical print volume with a diameter of 400 mm and a height of 1000 mm. The LDM extrusion system consists of a pressurized 5 L tank that stores the clay and feeds it into the extruder. Inside the extruder, a spiral screw transports the clay to the nozzle. The rotation of the screw is directly driven by a Nema 23 3N.M stepper motor (Massa Lombarda, Italy). Therefore, the amount of material extruded is influenced by both the analog parameters of clay consistency and air pressure, as well as the digital parameter of screw rotation, as well as the positioning of the nozzle. To enable the injection deposition in the substate bed, the commercially available nozzle with a 6 mm inner diameter circular die was modified. This was performed by adding a 100 mm long metal tube with a 5 mm inner diameter. The length of the extension tube is limited by the viscous drag of the extruded material and the risk of extension buckling when injected in the aggregate suspension. The length of the extension tube in turn limits the height of the printable object to 150 mm in a volume of a 460 × 295 × 200 mm mesh container that allows water evaporation for the clay body on all sides. The container is enveloped in a waterproof material during printing. The waterproof envelope is removed after printing to allow for the drying of the substate and the injected clay body (Figure 1).

**Figure 2:**
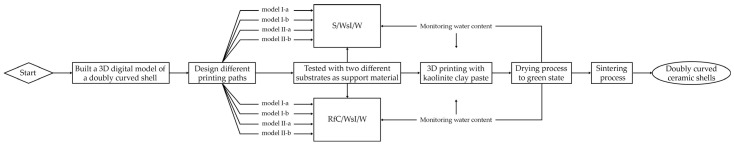
Flow chart: 3D printing process of four different models for doubly curved continuous shells in two different non-synthetic suspensions.

The steps behind the 3D printing of the objects are the following (Figure 2):

1. A single 3D doubly curved object is modeled.
2. The object is oriented and sliced in two different ways, resulting in total of four unique printing paths.
3. Each of the four printing paths is then executed in the two selected substrate mixtures S/WsI/W and RfC/WsI/W.
4. The water content of the mixtures and the resulting drying behavior of the objects in mixtures is noted, observed, and adjusted.
5. After complete drying, the objects are fired in a kiln at 1080 °C.
6. Final objects

### 2.2. Kaolinite Clay Paste

All objects and samples were produced with a stoneware clay body comprising 25% chamotte with grain sizes ranging from 0 to 0.5 mm (Table 1). 

**Figure 3:**
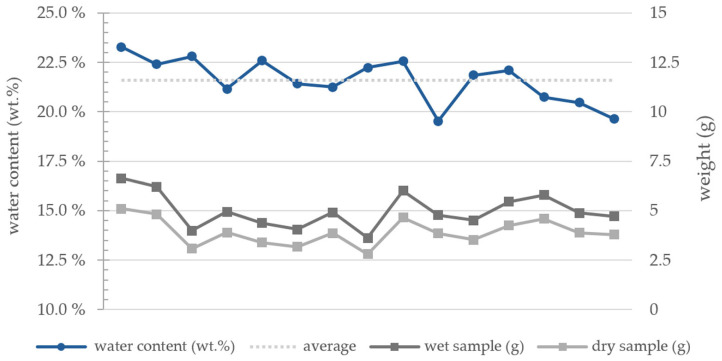
Water content (wt.%) of the clay paste compared to the weight of the wet and dry sample.

The clay paste was prepared manually by rehydrating the clay body to achieve the optimal consistency for extrusion. This process and various daytime temperatures resulted in a gradual fluctuation in plasticity during the experiments. Therefore, the fluctuations in the water content of the paste were measured by taking samples of equal amounts between the tests, ensuring the same motor rotations and tank air pressure. The wet sample weight was compared to the weight after drying at 100 °C for 24 h. The average water content of the used clay paste is 21.60 wt.%, with a fluctuation of ±1.88 wt.% (Figure 3).

**Figure 4:**
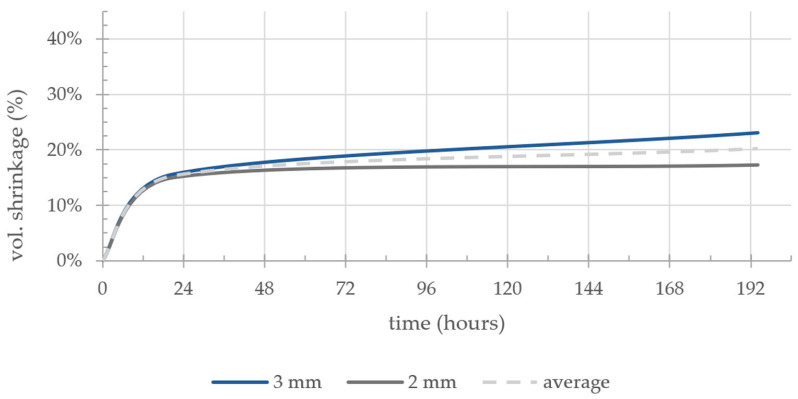
Volumetric shrinkage (vol.%) of the clay samples with 2 mm and 3 mm layer heights.

As information regarding the exact shrinkage of the clay body components is important for the composition of the substrate, the volumetric shrinkage was measured based on two groups of samples. As the influence of the layer thickness could not be predicted in relation to the shrinkage, the measurement was performed in two sample groups with 2 mm and 3 mm layer heights. The average dimensions of the produced samples in the wet state are 120 × 7.35 × 25 mm. The samples lost an average of 20.17% of their original volume after ambient drying for a week, with a difference of 5.89 vol.% in shrinkage between the 3 mm and 2 mm samples (Figure 4).

In the open air, the drying of the clay body took two (2) to five (5) days; in the explored process, the clay body dries after three (3) to seven (7) days depending on the substrate mixture and environmental temperature. For the study, the goal was to remove the geometry from the substrate in its leader hard state, when the clay is still plastic and has not stopped shrinking.

### 2.3. Particle Suspensions

In the case of injection 3D printing, the design of the support medium is of crucial importance. Through preliminary testing, factors that are relevant to a wider material spectrum of injection 3D printing and material specific challenges were defined. The support medium should have good holding capacity—the ability to maintain the position of injected materials despite gravity or dragging resulting through adhesion of the material to the nozzle. This can be achieved by using substrate materials with a high density or rigid particle structure. However, the support medium should also have high flowability, providing minimum resistance against the moving nozzle and to minimize the displacement of already deposited material by a passing nozzle. Gels and powders are support materials that combine these two characteristics. Considering the need for drying and the intention to obtain precise 3D-printed surfaces without compromising surface quality, only fine grain substrates are suitable in the case of clay injection 3D printing. Shrinkage occurring during the drying process leads to the necessity for a substate with a certain degree of plasticity and flowability that could accommodate the change in the position of the part to allow it to dry without cracking. 

#### 2.3.1. Initial Experiments

Considering these four factors of holding capacity, flowability, water absorption, and substrate plasticity, a range of substrate mixtures were evaluated to narrow the space of possible material combinations.

**Figure 5:**
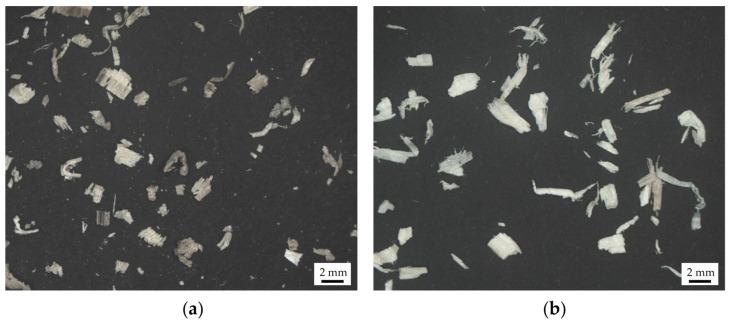
Wood shavings: (**a**) WsI—Hardwood shavings; (**b**) WsII—Softwood shavings.

**Figure 6:**
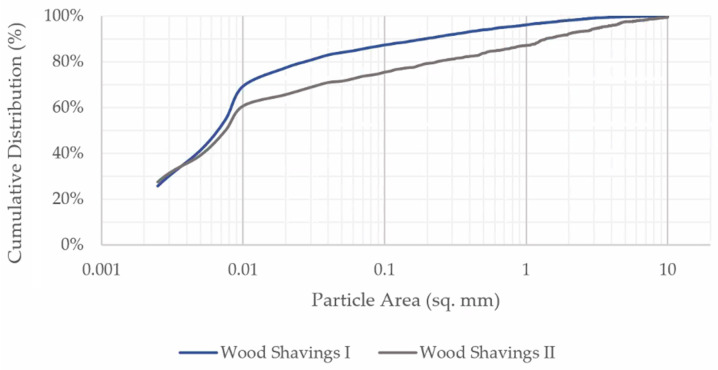
Cumulative particle distribution of WsI and WsII.

For the initial substrate explorations in each case, quartz sand (S) or refractory clay (RfC) were chosen as the ingredient that would provide holding capacity. The used quartz sand had a particle size range of 0.0 to 0.2 mm (Sakret, Berlin, Germany). Refractory calcined kaolin clay with grain size between 0.075 and 0.71 mm and a porosity of 10% was used in the substrate exploration and geometry production (Imerys Refractory Minerals, Molochite™, Cornwall, UK). Hardwood shavings form ash trees (Fraxinus excelsior) (WsI) and a mix of softwood shavings from spruce (Picea abies) and fir (Abies alba) trees (WsII) were evaluated for their ability to provide plasticity to the mixture. Both types of wood shavings were sieved through a 1.3 mm mesh. The particle geometry and distribution were analyzed using 2D image analysis (Figure 5a and Figure 6) [28]. WsI consisted of fine wood shavings with 69% of the particle area lying between 0.0025 and 0.01 mm^2^ (Figure 5b and Figure 6), while WsII had coarser particles with 61% of the particle area between 0.0025 and 0.01 mm^2^. The particle geometry of WsI is more circular, with an average aspect ratio of 1.6, while the particles of WsII have a rectangular profile with an average aspect ratio of 2 to 1.

Finally, talcum powder (T) and cotton flocs (C) were evaluated for an additional increase of mixture water absorption. The used cotton flocs consisted of white fibers with an average length of 0.35 mm.

These ingredients were mixed in three different ratios to explore the influence of each of the ingredients on the different factors. Due to the different densities and particle shapes between the wood and the sand/refractory clay in their dry state, a strong material segregation was observed. Therefore, in two groups of samples, water (W) was added to allow for a homogeneous mix, where trough capillary forces the sand stuck to the wood shavings to create small clumps. Table 2 shows the proportions of the individual initial samples.

Considering that the success of the sample stability depends mainly on the drying ability and the shrinkage, the compression ratio of the mixtures was examined. The tests were conducted in a way similar to the process described by Pokhrel et al. [29]. The tests were performed using a manual 2-coulum lab press and a cylinder matrix of 40 mm by 60 mm, where after filling the mold was hit 5 times with a rubber hammer to achieve the tap density. The samples were then gradually compressed up to 20 kN.

As summarized in Table 3, further tests were performed. To determine the water absorption capacity of the substate mixtures, the change in weight of the suspended gypsum cubes (15 × 20 × 15 mm) was measured. The gypsum cubes were first dried for 48 h at 50 °C, then soaked in water for 10 min, suspended in the prepared mixtures, and sealed in airtight bags. A separate substrate mixture with the same proportions was prepared for each time interval of 12, 24, 48, 72, and 120 h. The cubes were weighed in their dry, wet, and intermediate states to calculate the percentage of water absorbed by the different mixtures.

The flowability of the mixtures was evaluated through their angle of repose. The same volume of substrate at taping density was poured through a funnel to form a cone and then the angle between the cone’s surface and the horizontal base was measured. 

The holding capacity of the substrate was evaluated by printing a single line at different heights in the substrate and visually evaluating and comparing the results.

The best compression rate was observed in the mixtures with added water. This can be explained by the creation of small air pockets by the clumps of sand and wood shavings. There was no significant difference in compressibility between S and RfC observed in the mixtures containing either type of wood shavings. WsII exhibited better compressibility, which can be explained by the coarser particle composition, leading to higher porosity at taping density. Mixtures containing WsI exhibited better water absorption, which would lead to a more breathable substrate mixture that would allow for water from the embedded clay body to evaporate faster. The addition of water absorbing agents T and C did not improve the water absorption of WsII. Evaluating the angle of repose, it can be concluded that the flowability of S and RfC are similar. The two types of wood shavings have similar flowability behavior. However, when comparing sand mixtures containing each of the two types of wood shavings, S/WsI has a significantly better higher flowability. The flowability of RfC/WsI is lower than that for S/WsI. The addition of around a 15 vol.% of water significantly decreases the flowability of the mixtures, with both 3S/3WsI/W and 3Rfc/3WsI/W having similar angles of repose. When evaluating the line test, the best results were observed in the mixtures containing water. WsII exhibited a slightly better holding capacity compared to WsI, but due to the larger particle size led to larger indents in the sample surface, which could lead to a lower structural strength for the objects.

Comparing these results, the balance between holding capacity, water absorption, plasticity, and flowability proved to be suitable in both S/WsI/W and RfC/WsI/W mixtures. Therefore, the final geometries were printed in both mixtures to evaluate their potential.

### 2.4. Digital Modeling Strategies

**Figure 7:**
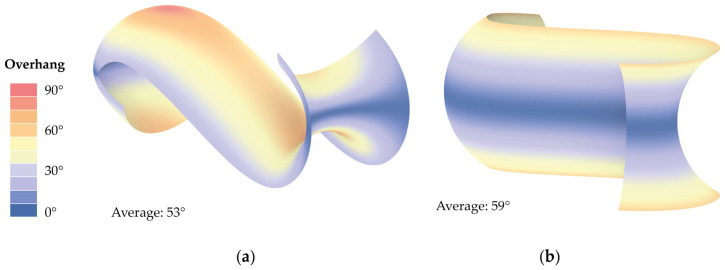
Overhang diagram: (**a**) Horizontal orientation (Model I); (**b**) Side orientation (Model II).

Modeling of the digital geometries and compiling of the machine instructions (G-code) was conducted inside the parametric modeling software Grasshopper 3D in Rhino 7 [30]. To test the examination setup for the production of complex-shaped ceramic components in the defined substrate mixtures, a doubly curved surface with an elongated S-shaped profile was designed. The main consideration for the design was to create a shape with overhangs that would otherwise not be printable using traditional LDM (Figure 7). Therefore, it is not possible to use this geometry directly as façade cladding, for example. The double curvature combined with the geometry folds into and onto itself, creating varying overhangs and internal cavities along its length. This geometry fitted both horizontally (Model I) and on its side (Model II) in the meshed substrate container of the experimental setup.

**Figure 8:**
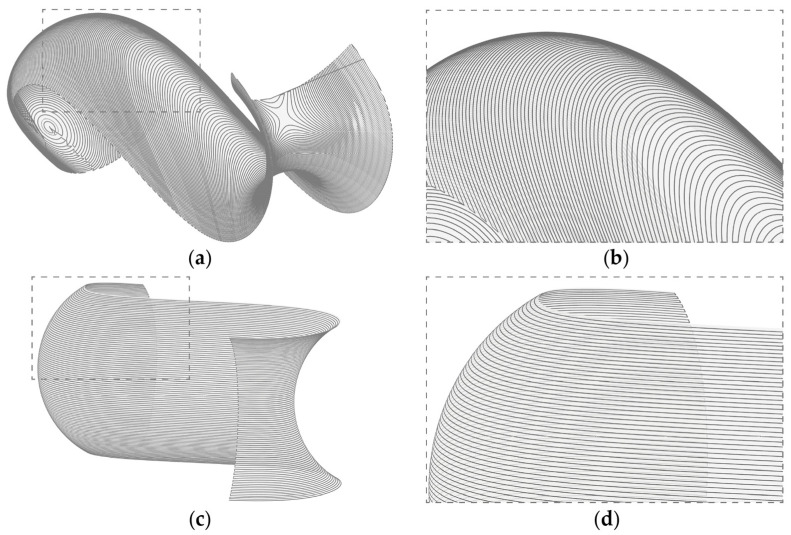
Printing Path: (**a**) Curvature adapted layers at −45° (Model I-a); (**b**) Detail of Model I-a; (**c**) Horizontal curvature adapted layers (Model II-a); (**d**) Detail of Model II-a.

A non-standard slicing was applied on the two different orientations to minimize travel paths, the layer staircase effect, and collisions of the nozzle with previously deposited material cf. [31]. Travel paths, where no material is extruded, are generally instances of the toolpath that should be minimized, as they lead to increased printing times and can lead to connection failures in the material body. Furthermore, in the case of injection 3D printing, they can also lead to shifting of the deposited material due to movement of the substrate. Due to the steep overhangs that were to be produced, utilizing a standard horizontal layer slicing with a constant layer height would have led to an acute staircase effect in the produced object [32]. The most affected layers being the ones generated on a curvature whose tangent is close to parallel to the slicing plane and where no layer overlapping takes place. That is why a curvature adaptive slicing was applied, where the profile curve was divided into equal length segments on which the slicing planes were projected, leading to parts of the geometry that have denser layers (Figure 8).

The horizontally oriented geometry was sliced at −45° and with curvature adapted layer distances to allow the object to mostly be printed without travel paths and without collisions with previously deposited material. The geometry oriented on its side was sliced with horizontal layers with curvature adapted distances (Table 4).

**Figure 9:**
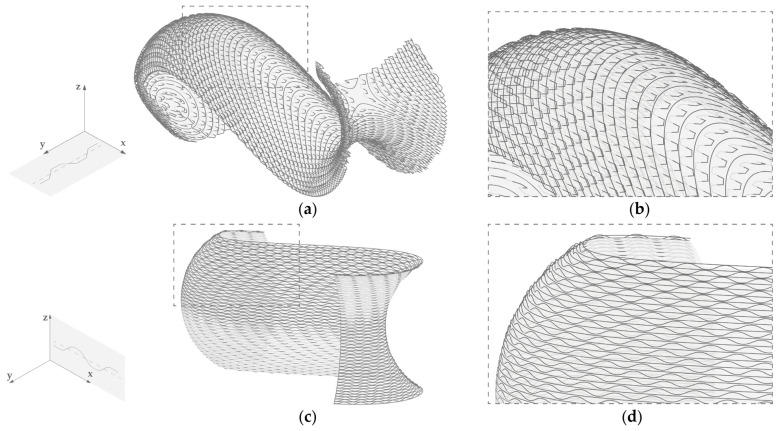
Printing path: (**a**) Curvature adapted layers at –45° with horizontal undulations (Model I-b); (**b**) Detail of Model I-b; (**c**) Horizontal curvature adapted layers with vertical undulations (Model II-b); (**d**) Detail of Model II-b; Coordinate system on the left show the addition of undulations (solid line) to the original toolpath (dashed line).

A 3D-printed object exhibits anisotropic material bond strength with the weakest bond being between the layers [33]. Considering the shrinkage of the deposited clay and the resistance force applied on the geometry by the substrate, cracking is mostly expected to occur between layers. The consistency of the clay paste for LDM has allowed for selective re-extrusion in previously deposited layers and has been shown to improve interlayer bond strength [34]. Based on this, two alternative geometries were produced, where undulations of the toolpath were introduced each second layer to allow the partial layer overlap of 2/3 of the layer width (Figure 9).

### 2.5. Injected 3D-Printed Clay and Sintering

To evaluate the effect of the substate mixture on the layer adhesion and bond strength a 3-point bending test was conducted. Samples were produced both in dry and wet mixtures and control samples were produced using traditional LDM. All sample groups were sanded, ensuring the plane-parallel top and bottom sides and were sintered at 1080 °C. The wood shavings underwent pyrolysis during sintering, leading to a rougher surface compared to the control samples. A color change was observed in the samples produced in S mixtures containing quartz sand, where some of the sand particles turned form brown to pinkish red, presumably due to the oxidation of impurities in the used quartz sand. After sintering, the samples had an average dimensions of 118 × 11.25 × 18.75 mm.

#### 2.5.1. Interlayer Bond Strength of Clay Samples in Green State

The substrate mixture had an influence on sample stability when the samples were in their green state by creating a film of dust and dry clay between the layers, leading to sample failure. The samples produced in the dry S/WsI and RfC/WsI mixtures exhibited weak layer adhesion and fell apart after being taken out of the mixture. Due to the porosity of the refractory clay, the water in the wet mixture was absorbed and led to instability of the green state samples, similar to the samples produced in the dry substrate mixtures. Some samples broke during the post-processing that was needed to equalize the sample geometry. To increase the layer strength and to be able to compare the effects of dry and wet mixtures, samples with lower layer height were produced in the dry S/WsI mixture. The failure rate was highest in the dry substrate mixtures printed with a 3 mm layer height, followed by the RfC/WsI/W samples that were printed in a mixture containing 15 vol.% water, which was mostly absorbed by the refractory clay (Table 5). A decrease in layer height from 3 mm to 2 mm decreased the failure rate of samples printed in the dry substrate (S/WsI) by 100%.

#### 2.5.2. Three-Point Bending Test

**Figure 10:**
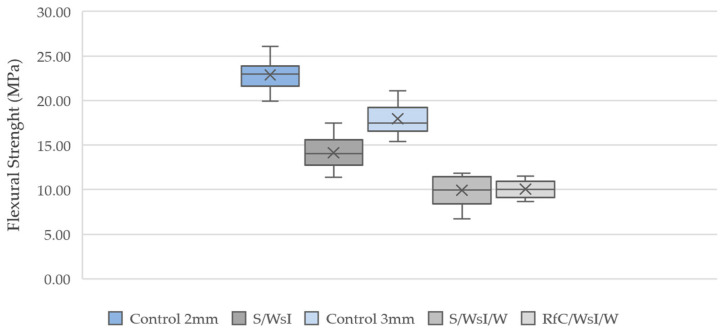
Box plot, 3-point bending test. The mean value is marked with X and the median is marked with a line inside of the box.

The 3-point bending tests were with 75 mm support spacing and 0.5 mm/min testing speed (Figure 10).

Overall, the substate samples exhibit lower flexural strength compared to the control samples. With a significant decrease of 38% in the 2 mm samples and a 44% decrease in the 3 mm samples. The wet substrate mixtures have similar average flexural strengths, 9.93 MPa for S/WsI/W and 10.5 MPa for the RfC/WsI/W, with RfC/WsI/W having a lower standard deviation. However, as the components could be used in the area of architectural cladding elements, where compressive load-bearing capacity is not as crucial compared to load-bearing elements, this reduction in load-bearing capacity is not initially considered to be an exclusion criterion for the production process. In addition, we will even discover an alternate application (cf. Section 2.5.4).

#### 2.5.3. Influence of Substrate on Layer Geometry

**Figure 11:**
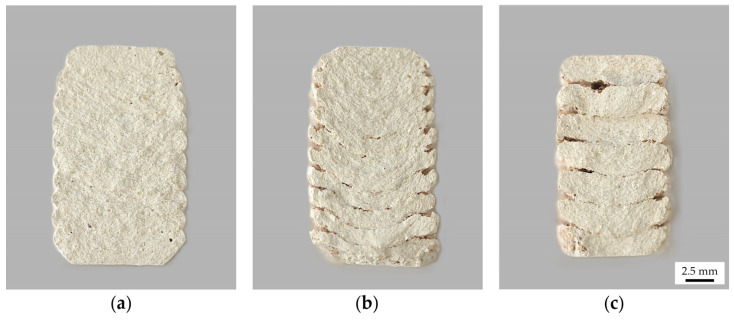
Fracture sections: (**a**) Control sample with 2 mm layer height; (**b**) S/WsI sample with 2 mm layer height; (**c**) S/WsI/W sample with 3 mm layer height. All samples have flat bottoms due to post-processing.

When observing the cross sections of the broken samples, deformations in the lowest layers were visible. Compared to the control samples, the layers became rounder at the bottom and the extruded clay was pressed down against the resistance of the substrate mixture (Figure 11). Similar to the observations made using the line test, this effect becomes weaker as the object is printed higher in the substrate. A plausible explanation for this effect is that the increase in material density in the lower levels of the substrate is due to the accumulation of material weight and the reduction of this effect in the following layers is due to the adherence of the next clay layer to the already printed geometry. In addition, the pressure against the substrate decreases as the layer build-up progresses because the material is already at the intended printing level.

#### 2.5.4. Additional Aspects

**Figure 12:**
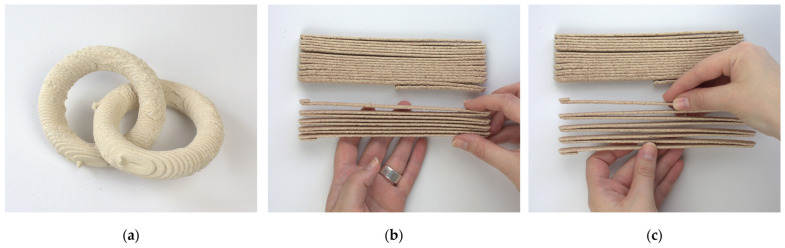
Additional structures: (**a**) Interconnected rings printed in RfC/WsI/W; (**b**,**c**) Springs in resting and extended states.

The substrates proved to also be suitable for the production of interconnected and movable parts, such as chains, which until this point could only be produced using Binder Jetting 3D printing (Figure 12a). Furthermore, the lowered binding ability of the samples in sand substrate mixtures (Section 2.5.1) could also be used to produce flexible structures (Figure 12b,c). The springs were printed vertically in the substrate with a layer height of 3 mm. Their dimensions in the resting state are 189 × 8 × 26 mm (100%), with elasticity along the vertical axis 189 × 8 × 410 of + 57.7% more, so (157.7%).

## 3. Results

**Figure 13:**
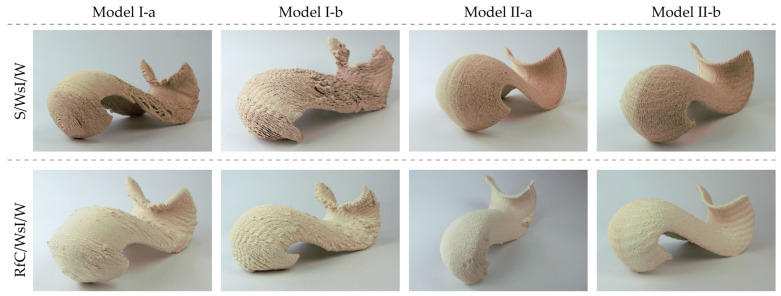
Perspective view of the various models in S/WsI/W and RfC WsI/W.

The tests were carried out in parallel with four different modeling strategies and in both S/WsI/W and RfC/WsI/W substrate mixtures (Figure 13).

**Figure 14:**
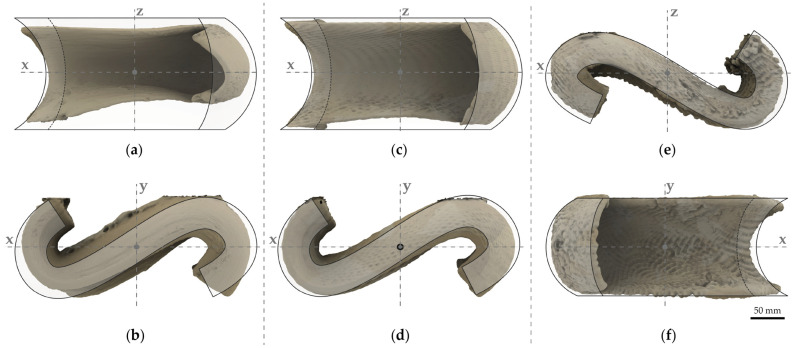
Frontal and top view of scanned 3D-printed geometries overlayed by the digital surface: (**a**,**b**) Model II-a in S/WsI/W with deformed geometry due to premature substate removal; (**c**,**d**) Model II-b in S/WsI/W; (**e**,**f**) Model I-b in S/WsI/W.

The examined substate mixtures and the drying procedure were adjusted during the production of the geometries due to cracking or deformation. The first difficulty was to evaluate the exact time between substrate drying and clay body drying. The first printing tests were removed from the substate after three (3) days of air drying at an approximate room temperature of 20–22 °C, which proved to be too early, as the clay body subsequently deformed, as seen in the 3D-scanned geometry (Figure 14a,b). The drying time for the geometries was increased successively to five (5) days, as it was observed that the clay body was dry enough to support its own weight without deforming, while not being as brittle as in a bone-dry state (Figure 14c–f).

Furthermore, the amount of added water to the substrate mixture was recognized as an essential factor in the production process. As clay is still a water-based medium, the quality of the clay body is also influenced by its ability to continue to absorb water. The balance between the moisture content in the clay substance and the substrate must be calibrated so that the clay body is not damaged through water re-absorption. As sand has lower porosity and does not absorb water, the water content of the S/WsI/W mixture was, on average, lowered from the initial 15.00 vol.% to 11.00 vol.% after disintegration of the clay paste was observed in the first attempts of printing the geometry. Due to the porosity and therefore better water absorption capacity of the refractory clay grains, the initial 15.00 vol.% of water was increased to 25.00 vol.% on average to allow for better interlayer material bonding. However, this led to the drying period being on average one (1) day longer in comparison to the sand mixture.

With regard to the printing results in the different substrate mixtures, it can be concluded that when utilizing injection 3D printing in substrate mixtures, a narrower layer height domain for successful object production has to be used. A lower layer height could provide an increase in flexural strength for geometries produced in the wet substrate mixtures, which could lead to flexural strengths that are in the range of the control samples. Despite the high failure rate in its green state, it can be concluded that the interlayer bond strength of the RfC/WsI/W samples was positively influenced during sintering, pointing to a more beneficial relationship between the substrate and the injected clay. Furthermore, as opposed to the samples produced in the sand mixture, the samples produced in refractory clay mixtures had no visible color change because of the incorporated particles. 

## 4. Discussion

### 4.1. Limitations of the Used Technology

It has to be noted that the used set-up, consisting of a delta 3D printer and a box, has its physical constraints. Firstly, the height of the printed geometry is limited by the height of the nozzle actuator. Secondly, the width and depth of the used substrate container have to allow for the free movement of the three diagonal arms within the container. To avoid collisions with the walls of the container, it has to be oversized, which in turn leads to the need for more substrate volume that is not fully utilized.

**Figure 15:**
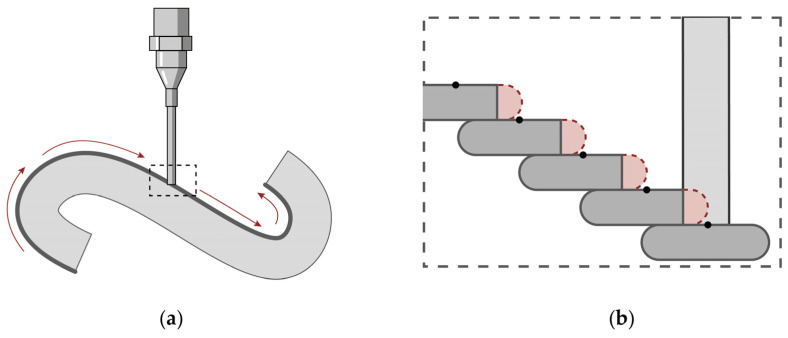
Scheme of printing sequence; red arrows showing the sequence of layers (**a**); Detail of the nozzle cutting deposited layers; red dotted areas representing removed material; black dots representing the middle of the toolpath (**b**).

Further, the effect of the static planar orientation of the nozzle on the material deposition of Model I has to be noted. In the slicing of Model I, the planes of slicing differ from the plane of the nozzle, leading to deposited material being cut by the nozzle when it has to perform downward printing paths (Figure 15). This led to a deformation on the shell in the middle section of Model I and contributed to cracks in the middle of the shell. This problem is not present in Model II, as the slicing planes are parallel to the plane of the nozzle.

Based on these observations, it can be presumed that injection 3D printing will be most feasible utilizing a robotic arm with 6 DOF, as it has the freedom of movement to avoid collisions, allowing for a smaller substrate holding container, while allowing for orientation of the nozzle normal to the surface that is to be produced.

### 4.2. Modeling Effects

**Figure 16:**
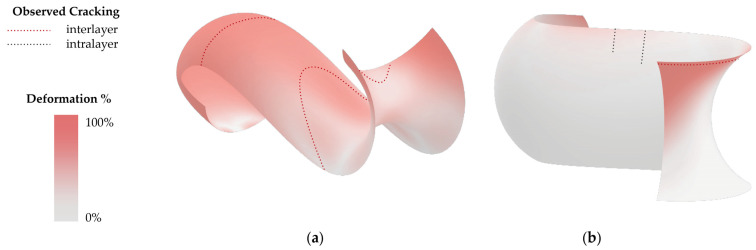
Deformation simulation and observed locations and types of cracks: (**a**) Model I; (**b**) Model II.

Two different approaches to the positional orientation of the doubly curved geometry were tested. It was found that the forces exerted by the dead weight of the clay body in the horizontal geometry have a much greater influence on the final shape of the 3D-printed clay body. This observation was predictable from the previous considerations on the application of force. In addition, there is a slightly unequal distribution of the drying phenomena because the substrate retains moisture longer in the lower areas of the container despite uniform distribution in the container, as was also experienced with the potential cracking against the course of the stratification in Model II (Figure 16b). The results show consistently stronger deformation of the horizontally supported components regardless of the type of layering used.

The position of the geometry in the substrate is also influenced by the shrinkage process. Since the clay bodies always shrink towards their relative center, the substrate is compressed differently in the horizontal or side-oriented component, which leads to different load conditions. In the case of the Model I, problems occur more frequently in the front part of the curve geometry, where the substrate is also kept moist for longer due to the inclusion under the geometry.

The overall better performance of Model II compared to Model I in terms of cracking can be attributed to the direction of shrinkage. The greatest shrinkage is anticipated and observed along the longitudinal axis of the geometry (Figure 14, *x*-axis), resulting in the most resistance force applied by the substrate along that direction. In Model I, the interlayer bond must resist this force, leading to frequent cracking in the middle of the geometry (Figure 16a). Conversely, in Model II, this force is applied parallel to the layer seams and is resisted by the intra-layer material bond, resulting in fewer cracks (Figure 16b).

The utilization of undulating self-intersecting paths had a clear benefit in Model I, where fewer cracks and deformations were observed in comparison to the geometry printed without printing path modifications. This beneficial effect can be attributed to both better interlayer bond strength cf. [34] and the overall higher material input (cf. printing time of the different models). The undulating printing path was also feasible in the side-oriented geometry, but the surface could also be printed successfully without path modifications.

It should also be added that the orientation of the model not only influences the print result but also affects the quantity of support substrate required. The substrate container only needs to be half-filled for the horizontally oriented Model I, while it must be completely filled with supporting substrate for Model II.

### 4.3. Differences between the Used Substrate Mixtures

**Figure 17:**
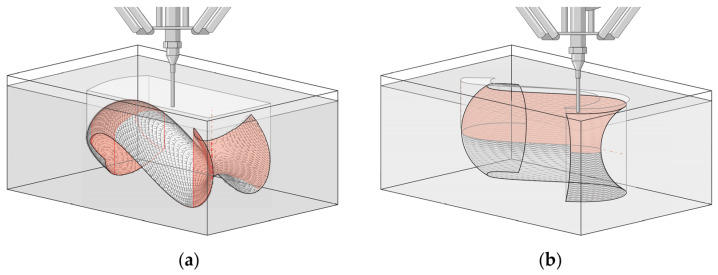
Scheme of affected areas (in red) due to displacement of the substrate: (**a**) For horizontally oriented surfaces; (**b**) For surfaces printed on their side.

Due to the increased plasticity of both mixtures, it was observed that when the nozzle moves through the substrate it became displaced. This led to already printed parts of the geometry being revealed. The resulting hole in the substrate is problematic for the parts of the geometries that fold onto themselves and enclose substrate in themselves (Figure 17). It should be noted that mass (substrate mixture) must first be displaced before the clay paste can be injected. Unlike gel suspensions that can self-level and return to their original shape, the substrate mixtures used retain their deformed shape after being displaced by the actuator, hindering the continuous printing process. This effect was observed equally strongly in the S/WsI/W and RfC/WsI/W mixtures, which is supported by the angle of repose results. In principle, the problem is more apparent in the horizontal orientation of Model I, which will ultimately be reflected in the results.

Similar to the observations made in the line tests and the samples used for the three-point bending test (Section 2.3.1 and Section 2.5.3), the underside surfaces of the green body were more strongly influenced by the substrate. The grains of the substrate create indentations in the surface of the clay body, which are most prominent after sintering, when the wood shavings have pyrolyzed. Geometries printed in the sand substrate mixtures have even stronger indentations due to the grains of sand that have not incorporated in the ceramic body after sintering. The geometries printed in refractory clay mixtures had fewer indentations due to a better bond between its grains and the clay body also resulting in more material inclusions. 

As mentioned above, another difference between S/WsI/W or RfC/WsI/W mixtures can be seen in the color effects of the surfaces. The sand mixture changes the color of the clay body after sintering, as the sand turns pinkish red, presumably due to oxidation, as it was not sintered without the exclusion of oxygen. The refractory clay substrate mixture does not cause any color changes.

A better interlayer material bond was observed in the samples printed in RfC/WsI/W, as no flexible geometries (see Section 2.5.4) could be produced in it. Even with sufficient layer height and comparable material settings as in the sand mixture, the refractory clay bonded with the clay particles during sintering.

Considering the reusability of the substrates, can be said that both S/WsI/W and RfC/WsI/W could be reused multiple times, with the additional step of sieving to remove some clay particles left by previous prints.

## 5. Conclusions

Using injection 3D printing with clay paste in non-synthetic particle suspensions, doubly curved continuous ceramic shells (326 × 150 × 147.5 mm) with average overhangs of 53° and 59° could successfully be produced. Four different modeling strategies were tested, proving that for the utilized set-up, a horizontal layering with additional material re-extrusion could produce a model without cracking that was close to the original digital model and with sufficient interlayer bonding strength. The feasibility of two non-synthetic reusable substrates consisting of a sand/refractory clay and wood shavings in a volumetric proportion of 1:1 as a suitable supporting medium was proven. Both of the tested substrate mixtures led to around a 41% reduction in the flexural strength of the samples. Overall, RfC/WsI/W compared to the S/WsI/W substrate mixture showed a slightly better performance due to its better effect on flexural strength of 10.5 MPa, fewer deformations, and better surface effects in the final geometries.

The reuse of both substrate types was feasible, with minor processing required to remove residual clay particles, ensuring sustainability in material usage. Adjustments in the drying procedures and substrate mixtures were essential to prevent cracking and deformation, with the optimal drying time for air-drying substrates (approximately 20–22 °C room temperature) identified as seven days to ensure structural integrity. It can be assumed that the use of a drying cabinet with even ventilation will speed up the process in the future. The need for precise control over the moisture content in the substrate mixtures was highlighted, as this balance is crucial for maintaining the quality of the clay body without re-absorption damage. The need for precise control over the moisture content in the substrate mixtures was highlighted, as this balance is crucial for maintaining the quality of the clay body without re-absorption damage.

Despite the success, further research is needed to refine the substrate mixture properties to reduce plasticity while enhancing elasticity and flowability without compromising its holding capacity. It also has to noted that the scalability of the process is not clear, as the production of larger elements requires a higher volume of substate, which in turn leads to a higher mass and compression effect on the printed geometries. The current setup, involving a delta 3D printer, faced limitations in height and movement, suggesting that a robotic arm with 6 DOF would potentially better suit the injection 3D printing process. This process can find application in the building sector for the production of bespoke facade cladding elements with an increased requirement for environmental performance and aesthetics, which are enabled through doubly curved ceramic geometries.

## Figures and Tables

**Table 1:** Technical data * of “Ateliermasse Weiß 2505” stoneware body.

Chemical Analysis	SiO_2_	Al_2_O_3_	Fe_2_O_3_	TiO_2_	CaO	MgO	K_2_O
	75.0%	19.5%	0.80%	1.4%	0.20%	0.30%	2.30%
Moisture				16.8 wt.%			

* Data retrieved from SIBELCO [27]. Data relating to the stoneware body refers to the unfired condition.

**Table 2:** Proportions of ingredients in substrate mixtures at taping volume.

Ingredients Mixture Code	Sand	Refractory Clay	Hardwood Shavings	Softwood Shavings	Cotton Flocks	Talcum Powder	Water
S	1						
RfC		1					
WsI			1				
WsII				1			
C					1		
T						1	
2S/5WsI	2		5				
2S/5WsII	2			5			
3S/4WsI	3		4				
3S/4WsII	3			4			
5S/2WsI	5		2				
5S/2WsII	5			2			
3S/3WsI/C	3		3		1		
3S/3WsII/C	3			3	1		
3S/3WsI/T	3		3			1	
3S/3WsII/T	3			3		1	
3S/3WsI/W	3		3				1
3S/3WsII/W	3			3			1
3RfC/4WsI		3	4				
3RfC/4WsII		3		4			

**Table 3:** Comparison of volume during compression, water absorption, and angle of repose.

Mixture Code	Volume % at:		Water Absorption %	Angle of Repose
	1 kN	20 kN		
S	100.00%	91.49%	26.37%	31.83°
RfC	83.33%	74.07%	29.80%	33.65°
WsI	56.62%	23.99%		49.33°
WsII	51.76%	15.71%		50.31°
Cf	38.39%	17.27%		
T	62.63%	32.91%		
2S/5WsI	73.39%	44.03%		
2S/5WsII	70.77%	44.08%		
3S/4WsI *	81.47%	53.97%	79.89%	36.53°
3S/4WsII *	75.39%	54.32%	56.95%	40.47°
5S/2WsI	94.24%	75.39%		
5S/2WsII	91.65%	74.25%		
3S/3WsI/C	75.37%	52.02%		
3S/3WsII/C *	77.20%	52.26%	57.19%	
3S/3WsI/T	78.74%	59.06%		
3S/3WsII/T *	70.95%	54.32%	50.17%	
3S/3WsI/W *	60.91%	38.92%		47.73°
3S/3WsII/W	54.37%	35.65%		
3RfC/4WsI *	82.12%	53.01%		40.02°
3RfC/4WsII	78.23%	53.29%		
3RfC/3WsI/W *				46.36°

* Substrates used for line tests.

**Table 4:** Printed geometries and parameters.

Model	Geometry	Dimensions L × W × H (mm)	Rotation of Slicing Planes	Layer Distance (mm)
Model I-a	S-horizontal	345 × 160 × 145	−45°	0.75–1.50
Model II-a	S-on side	345 × 145 × 160	0°	1.00–2.00
Model I-b	S-horizontal + undulations *	345 × 160 × 145	−45°	1.50
Model II-b	S-on side + undulations *	345 × 145 × 160	0°	1.00–2.00

* The + sign is used to describe the addition of undilations in the toolpath of the two original geomerties.

**Table 5:** Green state sample failure rate.

Group	Layer Height	Failure Rate
Control	3 mm	0%
S/WsI	3 mm	100%
RfC/WsI	3 mm	100%
S/WsI/W	3 mm	33%
RfC/WsI/W	3 mm	73%
Control	2 mm	0%
S/WsI	2 mm	0%

## Data Availability

The original contributions presented in the study are included in the article, further inquiries can be directed to the corresponding author.
